# Addressing missing values in routine health information system data: an evaluation of imputation methods using data from the Democratic Republic of the Congo during the COVID-19 pandemic

**DOI:** 10.1186/s12963-021-00274-z

**Published:** 2021-11-04

**Authors:** Shuo Feng, Celestin Hategeka, Karen Ann Grépin

**Affiliations:** 1 School of Public Health, University of Hong Kong, Pok Fu Lam, Hong Kong; 2 Harvard TH Chan School of Public Health, Harvard University, Boston, MA USA

**Keywords:** Missing data, Routine health information systems (RHIS), Health management information system (HMIS), Health services research, Low- and middle-income countries (LMICs), Multiple imputation

## Abstract

**Background:**

Poor data quality is limiting the use of data sourced from routine health information systems (RHIS), especially in low- and middle-income countries. An important component of this data quality issue comes from missing values, where health facilities, for a variety of reasons, fail to report to the central system.

**Methods:**

Using data from the health management information system in the Democratic Republic of the Congo and the advent of COVID-19 pandemic as an illustrative case study, we implemented seven commonly used imputation methods and evaluated their performance in terms of minimizing bias in imputed values and parameter estimates generated through subsequent analytical techniques, namely segmented regression, which is widely used in interrupted time series studies, and pre–post-comparisons through paired Wilcoxon rank-sum tests. We also examined the performance of these imputation methods under different missing mechanisms and tested their stability to changes in the data.

**Results:**

For regression analyses, there were no substantial differences found in the coefficient estimates generated from all methods except mean imputation and exclusion and interpolation when the data contained less than 20% missing values. However, as the missing proportion grew, *k*-NN started to produce biased estimates. Machine learning algorithms, i.e. missForest and *k*-NN, were also found to lack robustness to small changes in the data or consecutive missingness. On the other hand, multiple imputation methods generated the overall most unbiased estimates and were the most robust to all changes in data. They also produced smaller standard errors than single imputations. For pre–post-comparisons, all methods produced *p* values less than 0.01, regardless of the amount of missingness introduced, suggesting low sensitivity of Wilcoxon rank-sum tests to the imputation method used.

**Conclusions:**

We recommend the use of multiple imputation in addressing missing values in RHIS datasets and appropriate handling of data structure to minimize imputation standard errors. In cases where necessary computing resources are unavailable for multiple imputation, one may consider seasonal decomposition as the next best method. Mean imputation and exclusion and interpolation, however, always produced biased and misleading results in the subsequent analyses, and thus, their use in the handling of missing values should be discouraged.

**Supplementary Information:**

The online version contains supplementary material available at 10.1186/s12963-021-00274-z.

## Introduction

There is a growing interest in using data sourced from routine health information systems (RHIS) to monitor and evaluate the performance of health programmes and interventions, especially in low- and middle-income countries (LMICs). Such systems typically comprise data collected on a pre-defined set of health indicators from health facilities at regular time intervals. Globally, the leading RHIS platform is known as the District Health Information Software 2 (DHIS2), which is currently used in over 72 LMICs (https://www.dhis2.org/) [1]. However, poor data quality is limiting the use of data sourced from RHIS in some settings. Missing values are one of the most common and challenging components of poor data quality in RHIS [2] as their presence introduces uncertainty and ambiguity into the data. Also, missingness often undermines the statistical properties and the performance of estimators developed using the data, thus limiting trust in the results obtained using these data [3].

Strategies to address missing data are not novel topics in the health informatics literature. But while a great number of missing value imputation algorithms have been developed to address this challenge, there is an ongoing debate about which imputation methods are the best in particular scenarios. For example, Waljee et al*.* [4] have demonstrated the superiority of the local random forest method (e.g. *missForest*) in imputing missing laboratory values, while Hong and Lynn [5] have pointed out that the use of imputed variables from random forest-based approaches could lead to severely biased inference in a simulation study. Studies in other settings suggest that the results generated from multiple imputation are unbiased and could more closely mimic the true data [6, 7]. However, it is generally agreed that no imputation method should be seen as uniformly superior in all kinds of datasets [4, 8]. Indeed, the choice of imputation method is highly dependent on the structure of the data. Whether it is from a longitudinal study [9], patients’ health records [10], gene expressions [3], or another source will greatly affect the performance of imputation methods and thus inferences drawn from subsequent statistical analyses.

RHIS datasets share common patterns of missing values. Importantly, and unlike other commonly used health datasets, RHIS datasets tend to have missing values primarily on the dependent variable. For example, researchers often want to examine whether the number of health facility visits has gone up or down following a public health programme. In this case, it is not uncommon to find that only a small proportion of facilities had reported consistently over time, introducing a pattern of missingness that is non-trivial to exclude or impute. Also, given that some facilities are likely to report more consistently than others, simply excluding those facilities with missing values (i.e. listwise deletion) would not only eliminate a large proportion of the sample but also potentially introduce bias into the subsequent statistical estimators. Another approach is to keep all or most facilities but to generate imputed data for the time periods with missing values to fill in the holes; however, it is unclear how the missing values should be imputed in RHIS datasets.

A recent systematic review found that researchers are increasingly making use of RHIS data for research and evaluation purposes in LMICs [11]. The study also found that the most commonly used analytical technique was time series analysis to test or account for trends (35%), including 10% of studies that used interrupted time series (ITS) analysis. Geostatistical analyses (16%) and pre–post-comparisons (15%) were the next popular techniques, while other longitudinal analyses (13%), other cross-sectional analyses (12%), difference-in-difference (7%), and scenario analyses on cost-effectiveness (2%) were also employed. However, this review also pointed out that 75% of the research articles had no description of how missing data were managed in their studies. Among the 25% that did address missing values, simply excluding facilities based on certain exclusion criteria was the most common technique, and only a few studies (10%) attempted imputation or other strategies to handle missing values [11].

To date, there has been no evaluation of missing value imputation methods for data sourced from RHIS in LMICs, a gap this article aims to fill. Namely, we evaluate the performance of popular missing value imputation methods alongside commonly used analytical techniques employed by studies that involve RHIS datasets. Specifically, we use the advent of the COVID-19 pandemic in the Democratic Republic of the Congo (DRC) as an illustrative case study to test the performance of various imputation methods. To evaluate the imputation methods’ performance, we calculated both the bias between the imputed values versus true values and the bias between the parameters estimated from seven imputed datasets versus the true parameter estimates generated from complete datasets. In addition, we also examined whether the imputation methods were sensitive to different missing mechanisms, to small changes in the data, and to a different randomly selected starting point of the imputation algorithm. Based on our findings, we recommend strategies to handle missing values when using such data in future studies in the DRC and other similar international contexts.

The remainder of this paper is structured as follows. “Methods” section provides a detailed description of the data source, imputation methods, and analytical techniques used in our evaluation as well as a discussion around missing mechanisms, stability, and the criteria used to evaluate the performance of the imputation methods. “Results” section presents our findings about the evaluation of imputation methods’ performance, and finally, we discuss our findings, the generalizability of our results, and the limitations of this study in “Discussion” section followed by “Conclusions” section which providers recommendations to others on the choice of missing handling methods in RHIS datasets.

## Methods

### Study context and data source

This study grew out of a broader project that aims to investigate the impact of infectious disease outbreaks (i.e. Ebola and COVID-19) on the use of health services in the DRC using data from the national health management information system (HMIS), a DHIS2 enabled RHIS [12]. In the DRC’s HMIS, health facilities are expected to report the number of visits delivered each month using a standardized paper form. These forms are then transferred to the health zone office and are entered into a centralized database by a health professional. Each health facility is normally expected to report on a complete set of health indicators every month. We defined the lack of a report to the central system for a given indicator at a given health facility in a given month to be a missing value in our study.

This study context was selected partially because the DRC represents an interesting and challenging international context in which to test these techniques but also because members of the research team have been working closely with RHIS data in this setting for years, thus making it a convenient location to undertake this study; however, we believe the DRC’s HMIS shares many features with RHIS in other LMICs, in particular with those in Sub-Saharan Africa and those implementing DHIS2-enabled HMISs. The current HMIS system began its implementation in the DRC in 2014; however, only in 2017 it achieved a national-level scale [13]. From the entire sample of 18,138 facilities in the DRC, we identified 5510 facilities that had reported every month between January 2017 and October 2020. As the COVID-19 pandemic began in the DRC in March 2020, we considered all data before this month as pre-COVID-19 and data collected since the beginning of March 2020 until the end of October 2020 as after the onset of COVID-19 or during the pandemic.

### Imputation methods

In terms of the data imputation methods evaluated, we first selected the ones utilized in past RHIS studies, i.e. exclusion, interpolation, and mean imputation [11]. We also examined other algorithms that have been used extensively in imputing missing values, namely random forest, *k*-nearest neighbour (*k*-NN), seasonal decomposition, and multiple imputation, including both the default predictive mean matching (PMM) method and a 2-level Poisson that accounts for the fact that the HMIS dataset is both longitudinal in nature and made up of count data (examples by Waljee et al. [4] and Stekhoven and Bühlmann [14]). The simplest method, mean imputation, was also included for comparison purposes.

Imputation methods can be categorized as either a single imputation (SI) or a multiple imputation (MI) method, where a SI algorithm fills in each missing data point with only a single value. With only one guess at the missing value, SI tends to underestimate variance in the subsequent parameter estimates as the imputed value itself adds uncertainty [15]. In this study, we included four popular SI methods, namely simple mean imputation, exclusion and interpolation, *k*-NN, and seasonal decomposition. An MI algorithm, on the other hand, uses a repeated procedure to estimate each missing data point several times. As a result, instead of a single guess at the missing values, an MI method produces multiple imputed guesses which can be combined to establish a more accurate parameter estimation process in subsequent analyses by better addressing uncertainty in the dataset and in the missingness. The impact of the choice of a SI versus an MI method is further discussed in later sections.

Table 1 provides a summary of the seven imputation methods examined along with a brief technical description for each method. All analyses were conducted through statistical software R [16]. The codes for implementing these seven imputation methods are available in Additional file 1.

**Table 1:** Summary of imputation methods

Single or multiple imputation	Name of imputation method	Description	R package	Level of complexity to implement
SI	1. Mean imputation	Missing values are replaced with the average of the entire non-missing population in the same month	N/A	Easy
	2. Exclusion and interpolation	Firstly, any facilities with three or more consecutive missing monthly reports are excluded. Next, missing values in the remaining facilities are filled with interpolation	N/A	Easy
	3. Nonparametric missing value imputation using random forest (*missForest*)	*missForest* is a relatively new random forest-based method, which treats the variable with missing values as a dependent variable and regresses it against all the other variables in the dataset through a random forest model. This process is repeated iteratively, and in each step, the missing values are filled with a better prediction. The iteration stops when some threshold is met, i.e. when the changes in the imputed values between steps become small enough. This method is popular because of its ability to handle both categorical and numerical data, as well as very little manual parameter tuning required in the implementation [4]	*missForest* [14]	Moderate
	4. *k* Nearest neighbour (*k*-NN)	For each missing data point, the *k*-NN algorithm looks for the other *k* non-missing observations that are the most similar to the missing one, by comparing their distance measures. The missing data are then filled by a weighted average of the *k* neighbouring but non-missing observations, with the weights calculated based on their Euclidean distances to the missing data point. One difficulty in this method is the choice of *k*. In our study, we use the default number of *k* = 10 nearest neighbours, but the choice of *k* can be more carefully tuned through cross-validation [17]	*DMwR* [18]	Difficult: users are required to specify the parameter *k*
	5. Seasonal decomposition	Seasonal decomposition is tailored to the handling of missing values in time series data and can be summarized in three steps. Firstly, it identifies and removes the seasonal component from the original time series. Next, the missing imputation is performed on the deseasonalized series. Finally, the seasonal component is added back to reflect seasonality [19]	*ImputeTS* [19]	Easy
MI	Multiple imputation	Multiple imputation also treats the variable with missing values as a dependent variable and estimates it based on the rest of the variables. This estimation is repeated multiple times (*M* times) with a random component involved and being slightly different in each estimation to account for the uncertainty in the missing values. *M* datasets with slightly different estimations of the missing values are returned at the end of the estimation procedure, and taking an average across the *M* estimations yields an unbiased estimate of the missing values. The multiple imputation by chained equations (*mice*) implementation in R, in particular, enables an iterative estimation of missing values in multiple variables and provides flexibility in imputing both categorical and continuous variables [20]. The two methods listed below (i.e. methods 6 and 7) are both within the MI family	*mice* [21]	Moderate to difficult
	6. MI with predictive mean matching (PMM)	In each iteration of the *mice* procedure, each missing value is filled with the value of a donor, which is a complete data point whose predicted score from a fitted regression model is the closet to the predicted score of the missing data point [22]	*mice* [21]	Moderate
	7. MI with 2-level Poisson	In each iteration of the *mice* procedure, imputation is accomplished through a mixed effects Poisson model which accounts for the longitudinal structure and/or cluster membership of the data	*countimp* [23]	Difficult: Users need to understand the dataset structure

In the implementation of methods 3, 4, 6, and 7, we also included leads and lags with one time unit into the imputation, as recommended by Honaker and King [24], which includes the time series’ own history and future to help predict the missing time point of interest.

In general, *missForest* and *k*-NN are considered machine learning algorithms because they do not explicitly require the users to define how the prediction is taking place, whereas seasonal decomposition assumes certain distribution for the values.

### Analytical techniques

After generating imputed datasets, we then evaluated the performance of the seven imputation methods mentioned above through three most commonly used analytical methodologies in RHIS datasets as identified in a previous systematic review [11]. These methods are:

1. Simple and multiple linear regressions;
2. Segmented generalized linear regressions, which is the recommended technique to conduct ITS studies [25] and is widely used in evaluating health system quality improvement interventions when randomization is not possible [26];
3. Parametric group comparisons through paired *t* tests and nonparametric comparisons through paired Wilcoxon rank-sum tests, both of which are widely used in pre–post-comparison studies.

However, not all of the commonly used analytical methods are appropriate for RHIS datasets. Given the longitudinal data structure and the fact that these datasets comprise counts data, linear regressions (or ordinary least-squares regressions) are likely to be statistically inefficient. On the other hand, *t* tests require the underlying assumption of normally distributed data, which is not necessarily satisfied for RHIS datasets. Segmented regression with an appropriate distribution specified (typically a Poisson or negative binomial) is therefore the most appropriate technique for estimating the effect of interventions with RHIS datasets. As a result, this paper will focus on examining the performance of the seven imputation methods on both a mixed-effect segmented Poisson regression model and a pre–post-comparison through a paired Wilcoxon rank-sum test. An examination of the imputation methods’ performance using linear regressions and paired t tests is also included in Additional file 1: Figures S.5a through S.5e and Table S.1 for those interested in still using those methods, even though we do not recommend their use with RHIS datasets.

For regression analysis, in particular a mixed-effect Poisson model, the facility-level monthly total number of outpatient visits was used as the target variable and the incidence rate ratio (IRR) was estimated, with the following explanatory variables:

1. *Time*—a discrete variable counting the number of months elapsed since January 2017;
2. *COVID*—a binary variable indicating the presence of COVID-19 pandemic, i.e. 0 for January 2017 through February 2020, and 1 otherwise;
3. *Log Population*—a continuous variable capturing the log-transformed population size of the health zone where the facility is located;
4. *Facility type*—a categorical variable specifying the type of facility. Possible values are Hospital, Health Post, or Health Centre;
5. *Province*—a categorical variable was added to account for the 26 provinces in the DRC;
6. *Season*—a categorical variable to control for each of the 4 seasons as there are known to be seasonal variations in the use of health services in comparable settings [27].

For pre–post-comparisons, we conducted both parametric *t* tests (available in Additional file 1: Table S.1) and nonparametric Wilcoxon rank-sum tests on each of the imputed datasets to examine whether there were statistically significant differences in the mean number of outpatient visits before versus during the COVID-19 pandemic using paired *t* tests and to examine whether there was a location shift in the median number of outpatient visits before versus during the pandemic using paired Wilcoxon rank-sum tests. The number of monthly outpatient visits was selected as a good overall measure of health services utilization in DRC, and this context was chosen because a decrease in the use of health service was found in Kinshasa, DRC following the onset of the COVID-19 pandemic [28].

### Missing data mechanism

Before the imputation methods can be evaluated, it is important to first understand the missingness mechanism in the dataset. Missingness mechanisms are typically classified as (1) missing completely at random (MCAR), where the probability of being missing is totally random and does not depend on the value of any variables; (2) missing at random (MAR), where the missing values in the variable may depend on the known values of other variables in the data but not on the missing variable itself; and (3) missing not at random (MNAR), where the missingness of a variable could depend on the missing variable itself [29].

If data are believed to MNAR, it is generally recommended to improve the data quality by re-collecting data rather than using an imputation method because the missing pattern is not observed in the dataset [10, 30]. On the other hand, if the data are believed to be MCAR, i.e. the probability of a data point being missing is totally random and independent from any of the other variables, then a complete case analysis in which missing values are simply removed would generate unbiased results in subsequent statistical analyses [31]. If, however, the data are believed to be MAR, i.e. the missing pattern can be fully identified using the observed data, some algorithms can be applied to impute the missing values, resulting in a new complete dataset with imputed values. This new complete dataset can then be used to conduct further analyses using an appropriate analytical method.

To simulate a scenario where the RHIS data were missing at random, we inserted missing values into an HMIS dataset consisting of 5510 always-reporting facilities from the DRC’s HMIS as follows: the monthly total number of outpatient visits at time $i$ and for facility $j$ was set to missing depending on the facility $j$’s location (city and province), facility type (one of Hospital, Health Post, or Health Centre), time (the number of months elapsed since January 2017), season (a four-level categorical variable: 1 for January to March, 2 for April to June, 3 for July to September, and 4 for October to December), log population, and a binary indicator of the COVID-19 pandemic (0 for January 2017 through February 2020, and 1 otherwise), through the following equation:

$$\begin{aligned} {\text{logit}}\left( {{\mathbb{P}}\left( {{\text{visits}}_{ij} = {\text{missing}}} \right)} \right) & = \beta_{o} + \beta_{1} {\text{time}}_{i} + \beta_{2} {\text{season}}_{i} + \beta_{3} {\text{COVID}}_{i} \\ & \quad + \beta_{4} {\text{Facility Type}}_{j} + \beta_{5} {\text{Province}}_{j} \\ & \quad + \beta_{6} {\text{City}}_{j} + \beta_{7} \log \left( {{\text{Pop}}_{j} } \right) \\ \end{aligned}$$

The above logit model was fitted using the entire population of 18,318 facilities to capture missing patterns. We then applied this fitted model to the study population of 5510 always-reporting facilities, where the model assigned appropriate probability of each facility-month report being missing based on the patterns discovered in the entire population. Finally, six datasets with different proportions of missing values were constructed by calibrating the cut-off probability of being missing so that there was exactly *X*% of missing values inserted in each dataset, where *X* = 5, 10, 15, 20, 25, or 30.

### Consecutive missingness

Occasionally, facilities may consecutively miss making their monthly reports rather than following a pattern that would instead be considered MAR. Table 2 summarizes the number of facilities with no missing monthly reports, with missing reports but no consecutive missing reports, exactly two consecutive missing reports, exactly three consecutive missing reports, and at least four consecutive missing reports, respectively. We observed that there was a considerable number of facilities with at least four consecutive missing reports (4446 out of 18,138 facilities, or approximately 25%), which led us to also consider the stability of imputation methods in datasets with consecutive missing values. Specifically, we generated two additional datasets with 15% and 30% consecutive facility-month reports randomly set to missing.

**Table 2:** Summary of facilities with different consecutive missing patterns

Missing pattern	*N* (%)
No missing	5510 (30%)
Have missed reports, but no consecutive missing	5511 (30%)
Exactly 2 consecutive missing	1768 (10%)
Exactly 3 consecutive missing	903 (5%)
At least 4 consecutive missing	4446 (25%)
Total	18,318 (100%)

### Evaluation metrics

As previously discussed, we were interested in examining both the bias between the imputed values versus the true values and the bias between the parameters estimated using the imputed datasets versus the true estimations obtained using the complete datasets. To distinguish these two types of bias, we will refer to the bias and root-mean-square error (RMSE) resultant from a direct comparison between the imputed values and the true values as the “Crude Bias” and the “Crude RMSE”. Formally, we defined:

$$\begin{aligned} & {\text{Crude Bias}} = \frac{{\mathop \sum \nolimits_{i = 1}^{n} \left( {X_{i}^{{{\text{imputed}}}} - X_{i}^{{{\text{true}}}} } \right)}}{n}{\text{, and}} \\ & {\text{Crude RMSE}} = \frac{{\sqrt {\mathop \sum \nolimits_{i = 1}^{n} \left( {X_{i}^{{{\text{imputed}}}} - X_{i}^{{{\text{true}}}} } \right)^{2} } }}{n}. \\ \end{aligned}$$

Crude bias and crude RMSE are important in this context because many studies use RHIS to make direct examinations or make decisions solely on the imputed values themselves. For example, monthly reports concerning time series graphs are often directly examined and used to monitor the evolution of certain health-related indicators. Therefore, it is important to ensure minimal bias in the imputed values themselves. In addition to the crude bias, the second type of bias we wished to investigate is the difference between the coefficient estimates obtained using the imputed datasets versus the true estimates using the complete data. Analytical technologies such as ITS play a key role in the evaluation of health system interventions [26], so producing estimations consistent with the true ones is also critical to the evaluation of imputation methods.

Secondly, we also evaluated the performance of the seven missing handling methods by their sensitivity to different missingness mechanism (i.e. MAR and consecutive missing).

Lastly, it is important to note that RHIS databases are typically updated regularly. For example, in the DRC, there is a monthly update of the datasets that reflects the accretion of reports obtained from health facilities, including some that may have been submitted with a delay. These RHIS datasets are meant to be updated frequently, and hence, it is important to ensure consistency in the imputed values as well as the subsequent estimators obtained using these data from month to month. We therefore also tested each imputation method’s stability to minimal changes in the dataset (i.e. with only 2 months of data removed). In particular, we designed a scenario where the last 2 months (September and October 2020) were removed as well as a scenario where two random months of data (i.e. 2 months chosen randomly from the entire dataset of 46 months with ten replications) were removed. We compared the performance of each imputation method on the datasets generated under those two scenarios with its performance on the original dataset to evaluate the method’s stability. Besides that, we also repeated each imputation model on the original dataset but with another random starting point, as many numerical optimization algorithms are found to be starting-point dependent [32], and thus, the choice of starting point could potentially have an impact on the convergence and performance of the imputation method.

## Results

### Levels of missing data in the DRC’s HMIS dataset

**Fig. 1:**
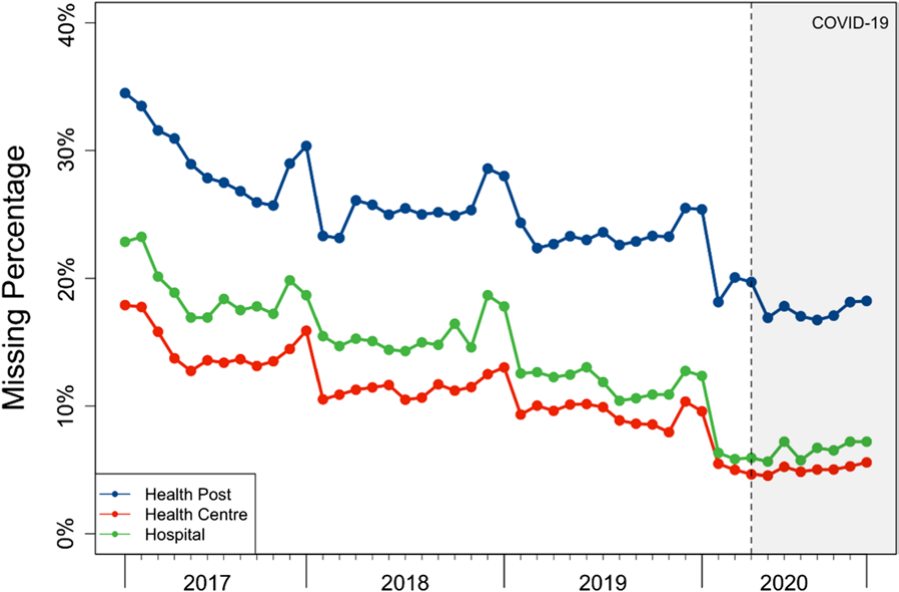
Missing percentages for monthly total visits by facility level

To provide context for this study, we first calculated the actual percentage of missing data in the DRC’s HMIS. In the original HMIS dataset, we observed a higher missing rate in health posts relative to health centres or hospitals (Fig. 1). However, the percentage of missing data has greatly decreased over time for all types of facilities. By 2020, there were only approximately 20% missing monthly reports in health posts and about 5% missing in health centres and hospitals. We note higher levels of missing data towards the end of each calendar year in all types of health facilities, which we speculated could be due to additional demands on those responsible for inputting facility-level reports during these periods for other activities; however, we are not able to fully validate this as the potential explanation.

In addition to the total number of outpatient visits, we also examined the levels of missing data for several other essential health services, including visits for common infectious diseases (uncomplicated pneumonia, uncomplicated diarrhoea, uncomplicated malaria, and rapid diagnostic tests for malaria), visits for maternal health services (antenatal consultations, institutional deliveries, and postnatal consultations), new diagnoses of non-communicable diseases (diabetes and hypertension), and vaccinations (DTP, BCG, OPV, and PVC-13). More details about the levels of missingness for these indicators can be found in Additional file 1: Figures S.1 to S.4b.

### Estimated coefficients under MAR assumption

**Fig. 2:**
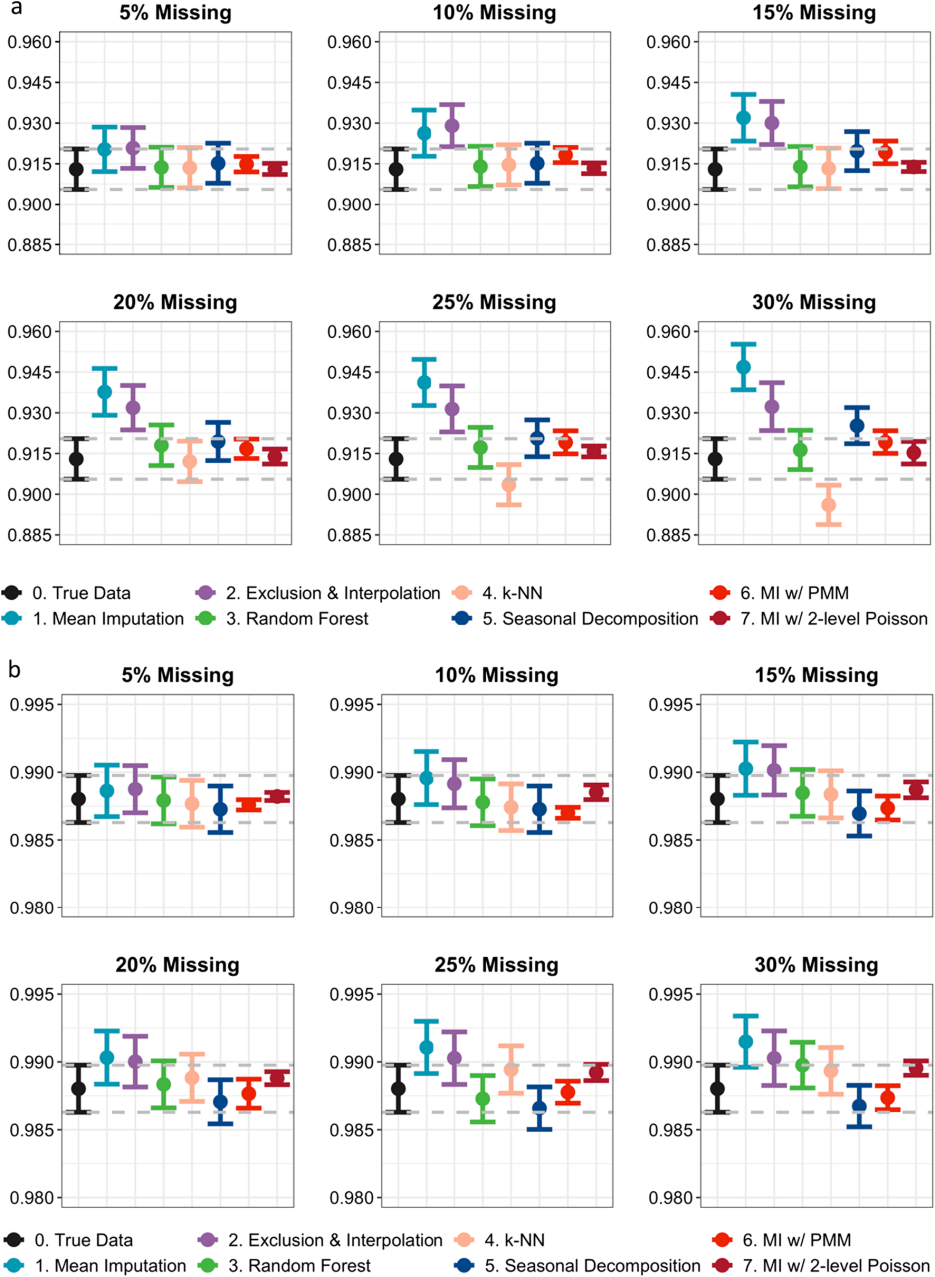
**a** Estimated level change in IRR with corresponding 95% confidence intervals. **b** Estimated trend change in IRR with corresponding 95% confidence intervals

Figure 2a and b shows the estimated level and trend change IRRs from segmented regressions of ITS. Their corresponding 95% confidence intervals were shown as error bars in the graphs. We observed from the graphs that the performance of all imputation methods—except mean imputation and exclusion and interpolation—was similar when there were only 5% missing values present in the data. As the missing proportions grew, however, the estimated coefficients and confidence intervals using imputed datasets started to deviate from the true estimates generated using the complete HMIS data. This separation among the estimates was found to be continuously aggravated as more missing data were inserted. On the other hand, the estimates based on the datasets generated using mean imputation or exclusion and imputation deviated from the true estimates immediately after even a very small number of missing values had been introduced. Both MI methods have produced smaller standard errors and thus narrower confidence intervals than all of the SI approaches regardless of the percentage of missingness introduced. In particular, MI with 2-level Poisson, which appropriately accounts for the data structure of RHIS datasets, has provided even narrower confidence intervals than the MI with default PMM.

### Crude bias and crude RMSE

**Fig. 3:**
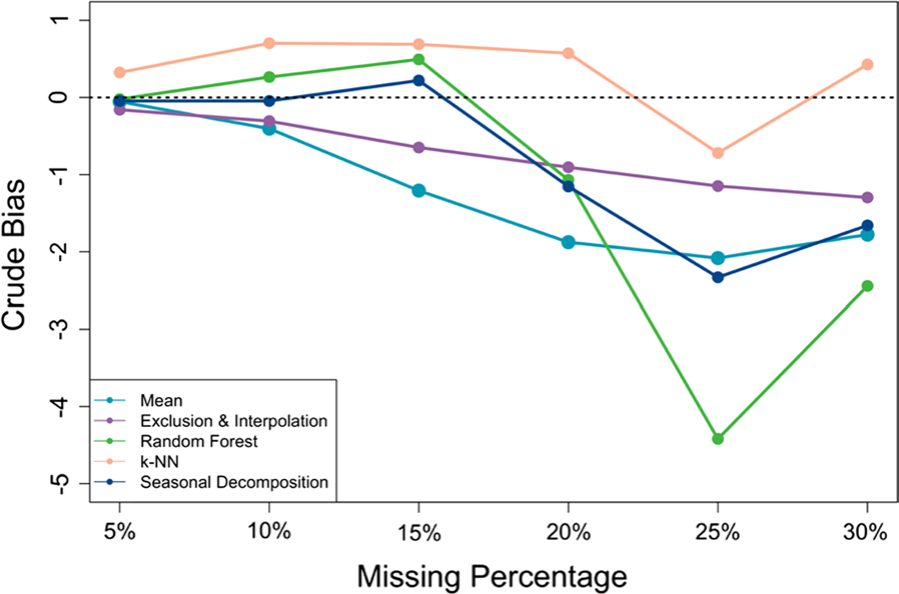
Crude bias between the imputed and true data with different missing percentages

**Fig. 4:**
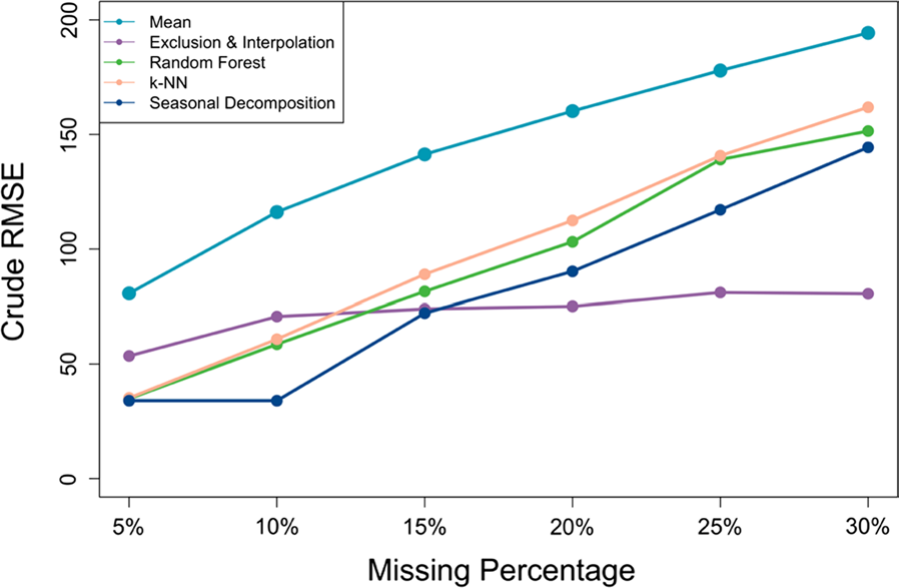
Crude RMSE between the imputed and true data with different missing percentages

Additionally, we examined how much the imputed values from SI methods deviated from the true complete data in terms of their crude bias (Fig. 3) and crude RMSE (Fig. 4), with different proportions of missing values inserted.

In our study, we observed that both the crude bias and crude RMSE grew as more missing values were introduced. With the same level of missing data, we found no material difference in the bias among the imputed values from different imputation methods, except *missForest* which has erroneously imputed values biased from the true numbers of visits when the missing proportion was relatively large (i.e. when more than 20% missing values present). In terms of crude RMSE, the data generated using mean imputation always had the largest crude RMSE, while that for the data generated using the exclusion and interpolation method was the most consistent across different missing proportions and was the smallest when the missing proportion was large. The other four SI imputation methods, however, were found not to be much different from each other regarding their crude RMSEs.

### Pre–post-comparisons

As the next most popular analytical techniques performed in RHIS datasets, pre–post-comparisons through paired Wilcoxon rank-sum tests were conducted to compare the numbers of monthly facility-level outpatient visits from January 2019 to October 2019 versus the numbers from January 2020 to October 2020, with the resultant *p* values provided in Table 3. For each individual facility, its monthly number of visits in 2019 was paired with its counterpart in 2020 (i.e. the number of visits from the same month in 2020). All estimated *p* values were found to be less than 0.01 as shown in Table 3.

**Table 3:** *p* Values obtained from comparing group medians using paired Wilcoxon rank-sum tests

Imputation method	5% missing	10% missing	15% missing	20% missing	25% missing	30% missing
Complete data	< 2.2E−16	< 2.2E−16	< 2.2E−16	< 2.2E−16	< 2.2E−16	< 2.2E−16
Mean	4.00E−14	5.71E−12	1.40E−07	4.59E−05	2.84E−04	6.13E−04
Exclusion and interpolation	< 2.2E−16	< 2.2E−16	< 2.2E−16	< 2.2E−16	< 2.2E−16	5.21E−15
Random forest	< 2.2E−16	5.60E−16	3.28E−15	3.99E−13	1.36E−05	8.09E−10
Multiple imputation	< 2.2E−16	< 2.2E−16	< 2.2E−16	5.36E−11	5.76E−09	4.70E−12
*k*-NN	< 2.2E−16	< 2.2E−16	2.15E−15	4.70E−14	7.23E−13	1.08E−08
Seasonal decomposition	2.51E−15	2.51E−15	3.74E−15	3.84E−11	1.10E−10	3.33E−10

### Consecutive missingness and stability

**Fig. 5:**
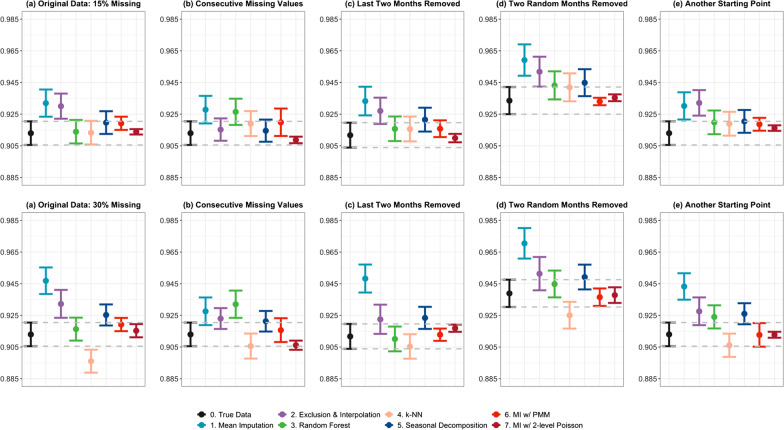
Estimated level change in IRRs and 95% CIs with missing values inserted under different scenarios

Figure 5 shows the estimated coefficients for the level change IRRs from segmented regressions with their corresponding 95% confidence intervals, on the datasets with 15% and 30% missing values inserted in different ways. Columns from left to right are estimates obtained from (a) original dataset with missing values inserted under the MAR assumption, (b) missing values inserted consecutively, (c) stability: dataset with last two months removed, (d) stability: dataset with two random months removed and with tenfold cross-validation, and (e) stability: original dataset with missing values inserted under the MAR assumption but the imputation algorithms started with a different starting point.

As can be seen from Fig. 5, besides mean imputation and exclusion and interpolation which always produced biased results, the datasets imputed using the two machine learning algorithms, i.e. *missForest* and *k*-NN, were found to be the most vulnerable to changes in the data, such as missing values inserted differently (consecutive missing), or minimal changes in the data (2 months data points removed). Seasonal decomposition produced consistent estimations only when the missing proportion was not large. Both types of MI algorithms were the most stable to all kinds of changes in the data. Also, whether the missing values were inserted under MAR or consecutive missing had little impact on all imputation methods other than *missForest* and *k*-NN.

The behaviour for the estimated trend changes was identical to the one shown in Fig. 5, which is also provided in Additional file 1: Figure S.6.

## Discussion

In this study, we observed a growing deviation between the regression coefficient estimates using the imputed datasets and the true estimates obtained using the complete data as more missing values were introduced. Specifically, with no more than 20% missing data inserted, all imputation methods, except mean imputation and exclusion and interpolation, generated accurate level and trend change estimates for segmented regressions (Fig. 2a and b). As the missing percentage grew, estimates from the datasets imputed using *missForest*, both types of MI, and seasonal decomposition still maintained a good level of accuracy, while *k*-NN started to produce severely biased estimates, especially in estimating the level change IRRs. When there were at least 30% missing values present in the data, estimates produced from all methods started to deviate from the true values. MI methods generated the overall most unbiased estimates for both level and trend change IRRs, aligning with previous findings in the literature by Myers [7] and Coffman et al. [32]. In addition, compared with the other five SI methods, both types of MI methods always produced much smaller standard errors, and thus narrower confidence intervals. The standard errors from estimates generated using the MI method that incorporated 2-level Poisson model, which in particular accounted for the longitudinal data structure and the nature of counts data, were even smaller (i.e. narrower CIs) than MI with the default PMM method, which emphasizes the importance of an appropriate handling of the data structure in producing smaller standard errors.

Among the five SI methods, besides from the comparison between regression estimates, we observed no substantial difference in their crude bias when the missing proportion was relatively small, i.e. when less than 20% missing values present in the data (Fig. 3). However, as the missing percentage grew, datasets imputed using *missForest* quickly became the most biased. This finding is consistent with previous evidence that implementing individual tree estimation through *missForest* could systematically lead to biased estimates, especially for non-normal, skewed data such as the count data that we had in our study [5]. Further, we found that the crude bias in imputed values became more aggravated as more missing values were introduced into the data. On the other hand, *k*-NN has consistently generated imputed values with the least crude bias, even when the proportion of missing was large; however, the estimators constructed using such an imputed dataset were not guaranteed to be unbiased. In fact, our study suggests that *k*-NN could lead to a more biased and unstable level change estimates than any other methods when the missing proportion was large as shown in Fig. 2a. For crude RMSE, while the data generated using mean imputation always had the largest RMSE (Fig. 4), the exclusion and interpolation approach outperformed all other imputation methods when the missing proportion was large. But this merit could be attributable to the fact that this method had already excluded those facilities suffering the most from missing values, and its RMSE was calculated solely based on the subpopulation of the most consistently reporting facilities. Lastly, the data imputed using seasonal decomposition produced the least RMSE when there were few missing values and outperformed the other imputation methods, except exclusion and interpolation, as the missing percentage grew. We otherwise observed no material difference across the methods.

For pre–post-comparison, we found all methods to produce a *p* value less than 0.01, regardless of the amount of missingness introduced, suggesting low sensitivity of paired Wilcoxon rank-sum tests to imputation methods.

In terms of stability, similar to a study conducted by Kokla et al*.* [33], the performance of *k*-NN in such complex datasets has again proven to be very unstable. The other machine learning algorithm, *missForest*, was also highly sensitive to small changes in the data or a different starting point, especially when the missing proportion was large (Fig. 5). Although *missForest* appeared to show good consistency in coefficient estimates in the initial MAR scenario (Fig. 2a), this lack of stability makes it as well as *k*-NN less ideal to use, especially when the missing proportion is large. On the other hand, both types of MI algorithms were found to be the most robust to all changes in the data. We also verified that whether the missing values were inserted under the MAR assumption or inserted consecutively turned out very little impact on all imputation methods aside from *missForest* and *k*-NN, both of which performed relatively poorly.

In practice, exclusion and interpolation is the most widely used method to deal with missing values among public health researchers who work closely with RHIS data [11]. Our study, however, suggests that the data imputed using this method can potentially lead to severely biased estimates and thus incorrect inference. Instead, the other statistically reliable but simple-to-implement imputation methods, especially the use of multiple imputation, should be encouraged. These methods can be implemented easily through existing packages from various statistical software, including the freely available software—R [16].

Though our study has shown both MI methods to outperform all other SI imputation methods because of its consistency across varying levels of missingness as well as its stability to all types of changes in the data, it may be challenging to implement MI in RHIS datasets due to its lengthy computing time. RHIS datasets typically cover a considerable number of facilities, and the number of observations is multiplied by the number of time periods for which the reports are collected. This massive amount of data, as well as the nature of multiple imputation to repeat the imputation algorithm several times, increases the computing time exponentially. This issue may be particularly challenging under LMIC settings where more limited computing resources may be available. In the scenario where the computing resources required for MI are unavailable, we recommend seasonal decomposition as the next best method to use, although the researchers must be cautious when the missing proportions are large as this method is observed to be relatively unstable to the changes in the data when the missing proportion is not small.

We believe our study can be well generalized to RHIS in other LMICs. Firstly, the DRC is a low-income country with a relatively weak health system [34]. In particular, the inefficiencies in the DRC’s health systems, including the failure to provide complete and consistent facility-level reports and human errors introduced by handling and transferring all the paper reports manually, are likely to be observed in many other LMICs. Also, among all the LMICs, the DRC is experiencing one of the most severe data quality issues as we illustrated in our study. The DRC’s HMIS has relatively high levels of missingness among LMICs whose health systems had been evaluated for their completeness in the literature. For example, Rwanda has a similar national HMIS but has been switched from paper forms to electronic HMIS since 2008. By 2012, there was no more than 5% missing reports for key indicators including general clinical visits, maternal health services, and vaccinations in its HMIS [35].

This study has several limitations. Firstly, we only included in our study those facilities that had reported every month between January 2017 and October 2020, and this study population (5510 facilities) is a small subset of the entire population (18,138 facilities) in DRC. Additionally, we considered the “true” estimates in this study as the ones that would have been obtained using the subpopulation of those complete (i.e. always-reporting) facilities. Indeed, these true estimates obtained using this subpopulation may not be representative of the entire population of 18,138 facilities. The entire population could be further examined to confirm the generalizability of our findings. It would also be interesting to validate externally whether our results generalize to the RHIS data in another country. Also, we only considered regression analyses and pre–post-comparisons in our analysis. While these three methodologies are among the most commonly used methods to analyse RHIS data [11], it is important to note the growing prevalence of machine learning techniques in the use of health data. Future research could also examine whether machine learning or deep learning algorithms developed based on RHIS datasets imputed by different missing value imputation methods would lead to similar conclusions.

## Conclusion

As Brock et al. [8] have pointed out, no imputation method should be seen uniformly superior in all kinds of datasets. Consistent with this message, our study finds that the performance of imputation methods based on RHIS data varies from other data contexts. Specifically, when the missing proportion was relatively low (i.e. less than 20%), we did not observe any substantial differences in the coefficient estimates generated from the five imputation methods evaluated (all except mean imputation and exclusion and interpolation), while both MI methods consistently outperformed all SI techniques due to a remarkably smaller standard error. As the missing proportion grew larger (i.e. when at least 20% missing values present), *k*-NN started to produce biased estimates. When the dataset is largely contaminated with missing values, i.e. when at least 30% missing values were inserted, all methods performed relatively poorly. Machine learning algorithms, i.e. *missForest* and *k*-NN, were also found to lack stability to small changes in the data or to consecutive missingness.

In terms of pre–post-comparisons, while we do not encourage the use of such direct comparisons, we found that the true dataset as well as the datasets imputed by any of the seven imputation methods all led to the same conclusion that rejects the null hypothesis of no difference in the monthly number of outpatient visits between pre- and during COVID-19 at a 95% significance level through paired Wilcoxon rank-sum tests, suggesting low sensitivity of paired Wilcoxon rank-sum tests to imputation methods.

We therefore recommend the use of MI methods in addressing missing values in RHIS datasets. We also recommend that, where possible, researchers should understand and account for the overall data structure in handling missing values (e.g. through specifying a Poisson model in the MI imputation process for counts data) to minimize the standard errors resultant from imputations. However, in cases where necessary computing resources are unavailable for multiple imputation, and where the missing proportion is relatively low, one may consider seasonal decomposition as the next best method to use. Mean imputation and exclusion and interpolation, on the other hand, always produced the most biased and misleading results in the subsequent analytical techniques, and thus, their use in handling missing values for RHIS data should be discouraged.

## Supplementary Information

**Additional file 1: Figure S.1.** Missing percentages for common infectious diseases clinical visits. **Figure S.2.** Missing percentages for the first antenatal consultation, institutional deliveries, and first postnatal consultation. **Figure S.3a.** Missing percentages for NCDs (diabetes and hypertension) new diagnoses. **Figure S.3b.** Missing percentages for diabetes-related visits by facility level. **Figure S.4a.** Missing percentages for vaccinations (DTP, BCG, OPV, and PVC-13). **Figure S.4b.** Missing percentages for DTP vaccination visits by facility level. **Figure S.5a.** Estimated coefficients with 95% C.I.s for variable Time from linear regression. **Figure S.5b.** Estimated coefficients with 95% C.I.s for variable log(Population) from linear regression. **Figure S.5c.** Estimated coefficients with 95% C.I.s for the binary variable COVID from linear regression. **Figure S.5d.** Estimated coefficients with 95% C.I.s for variable Facility Type: Hospital from linear regression. **Figure S.5e.** Estimated coefficients with 95% C.I.s for variable Facility Type: Health Post from linear regression. **Figure S.6.** Estimated trend change IRRs and 95% C.I.s with missing values inserted under different scenarios. **Table S.1.** *t* values obtained from comparing group means using paired *t* tests.

## Data Availability

We received authorization from the Ministry of Public Health to use these data to evaluate the impact of the pandemic on health service utilization. However, the dataset is not publicly available and researchers who wish to use these data are required to also obtain authorization from the Ministry of Public Health at the DRC (edited).

## Abbreviations

DHIS2: District Health Information Software 2
DRC: Democratic Republic of the Congo
HMIS: Health management information system
ITS: Interrupted time series
*k*-NN: *k-*Nearest neighbour
LMICs: Low- and middle-income countries
MAR: Missing at random
MCAR: Missing completely at random
MI: Multiple imputation
*mice*: Multiple imputation by chained equations
*missForest*: Nonparametric missing value imputation using random forest
MNAR: Missing not at random
PMM: Predictive mean matching
RHIS: Routine health information systems
RMSE: Root-mean-square error
SI: Single imputation
