# Supplementary material for: Addressing missing values in routine health information system data: an evaluation of imputation methods using data from the Democratic Republic of the Congo during the COVID-19 pandemic
Source: Popul Health Metr. 2021 Nov 4;19:44. doi: 10.1186/s12963-021-00274-z (PMC8567342; doi:10.1186/s12963-021-00274-z)
Supplement: Supplementary file 1 — Additional file 1: Figure S.1. Missing percentages for common infectious diseases clinical visits. Figure S.2. Missing percentages for the first antenatal consultation, institutional deliveries, and first postnatal consultation. Figure S.3a. Missing percentages for NCDs (diabetes and hypertension) new diagnoses. Figure S.3b. Missing percentages for diabetes-related visits by facility level. Figure S.4a. Missing percentages for vaccinations (DTP, BCG, OPV, and PVC-13). Figure S.4b. Missing percentages for DTP vaccination visits by facility level. Figure S.5a. Estimated coefficients with 95% C.I.s for variable Time from linear regression. Figure S.5b. Estimated coefficients with 95% C.I.s for variable log(Population) from linear regression. Figure S.5c. Estimated coefficients with 95% C.I.s for the binary variable COVID from linear regression. Figure S.5d. Estimated coefficients with 95% C.I.s for variable Facility Type: Hospital from linear regression. Figure S.5e. Estimated coefficients with 95% C.I.s for variable Facility Type: Health Post from linear regression. Figure S.6. Estimated trend change IRRs and 95% C.I.s with missing values inserted under different scenarios. Table S.1. t values obtained from comparing group means using paired t tests. [file 12963_2021_274_MOESM1_ESM.docx]

**Levels of Missing Data for Essential Health Service Visits**

There have been clear improvements in terms of reporting quality and consistency over time for the common infectious diseases (Additional file 1: Figure S.1) as well as the maternal health-related indicators (Additional file 1: Figure S.2), with only approximately 20% to 30% missing facility-month reports by 2020.

Additional file 1: Figure S.1: Missing percentages for common infectious diseases clinical visits


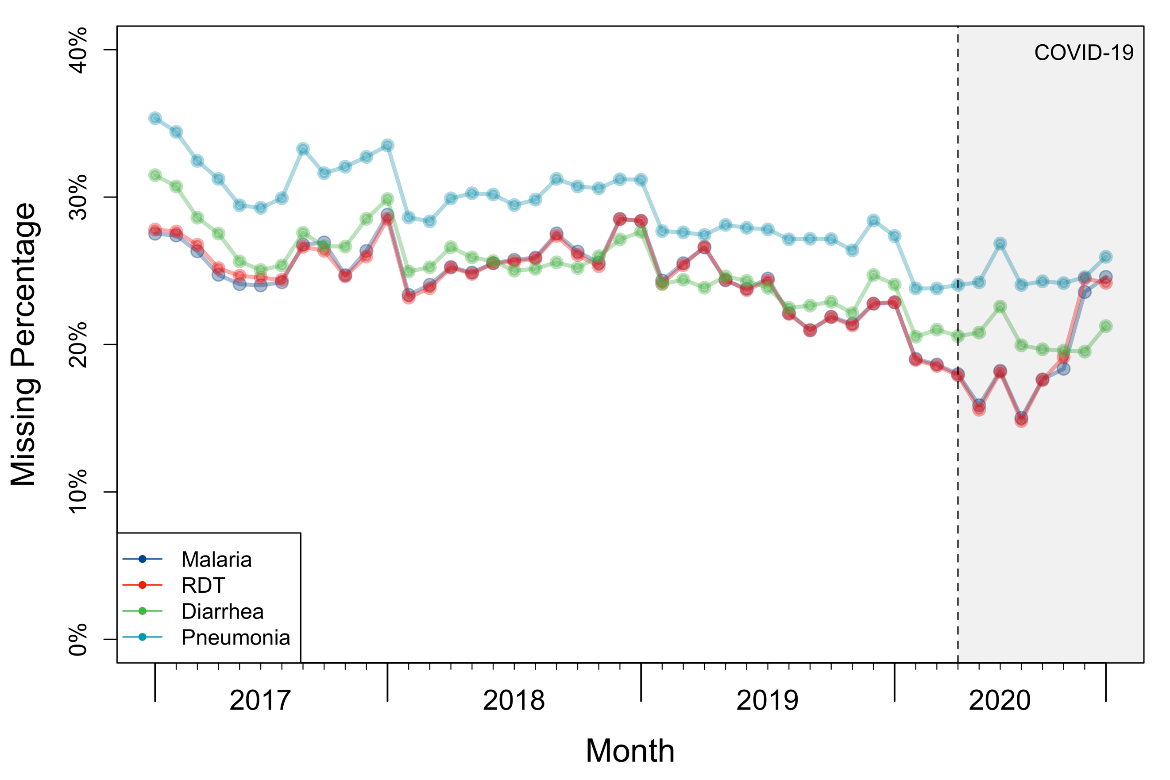


Additional file 1: Figure S.2: Missing percentages for the first antenatal consultation, institutional deliveries, and first postnatal consultation


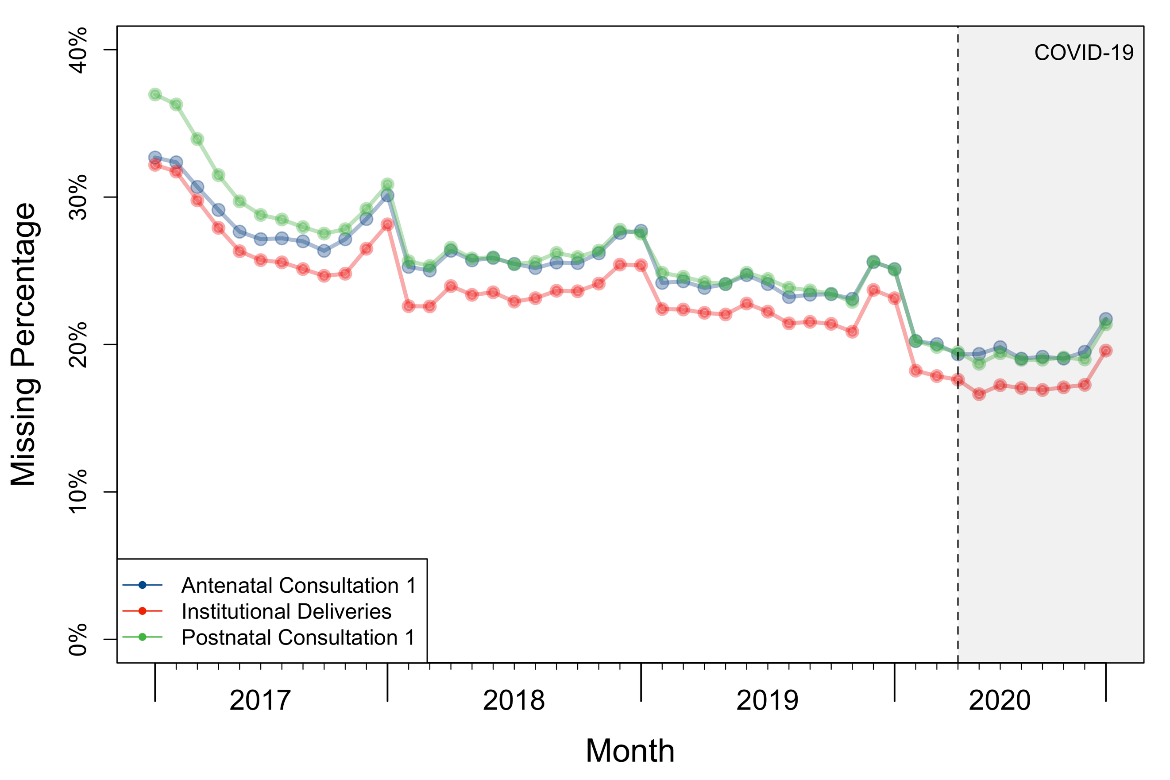


In terms of non-communicable diseases (NCDs) and vaccinations, we observed hospitals were always the major source of NCD visits in DRC (Figure S.3b), while most of the vaccinations took place in health facilities but not hospitals (Figure S.4b). The data quality has only been improved in hospitals for NCDs and in health centres for vaccinations, but not the other types of facilities.

Additional file 1: Figure S.3a: Missing percentages for NCDs (diabetes and hypertension) new diagnoses


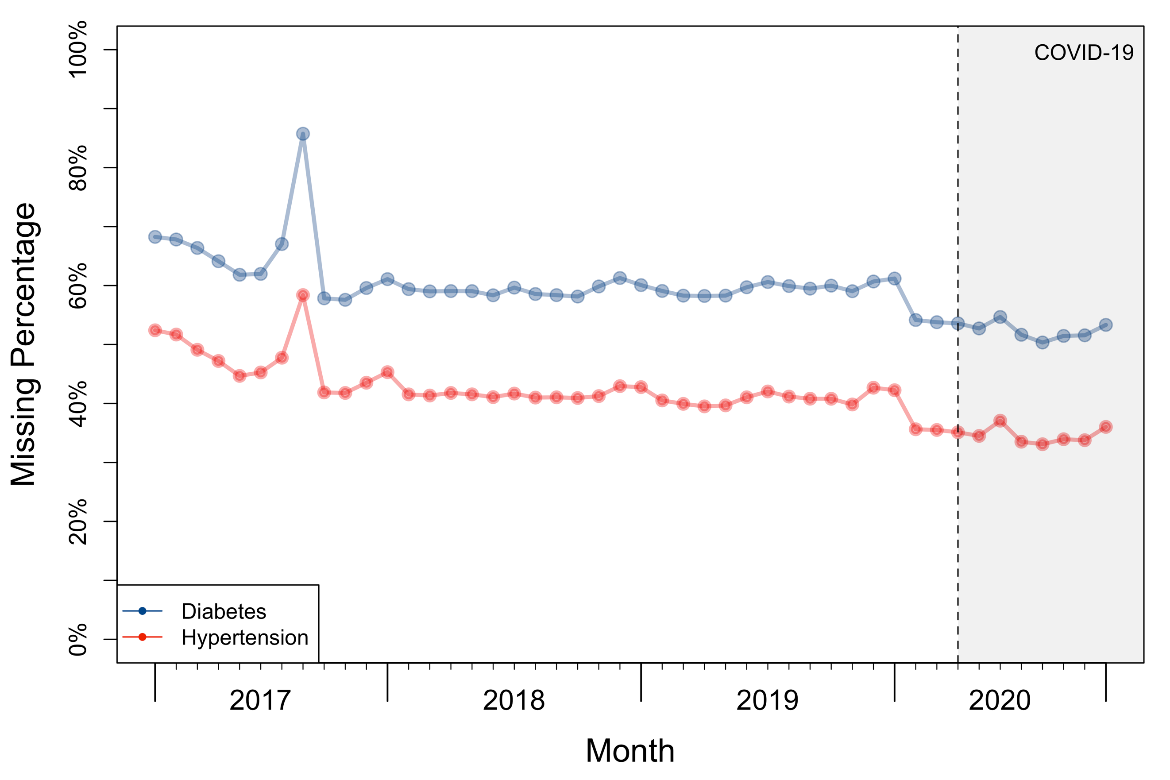


Additional file 1: Figure S.3b: Missing percentages for diabetes-related visits by facility level


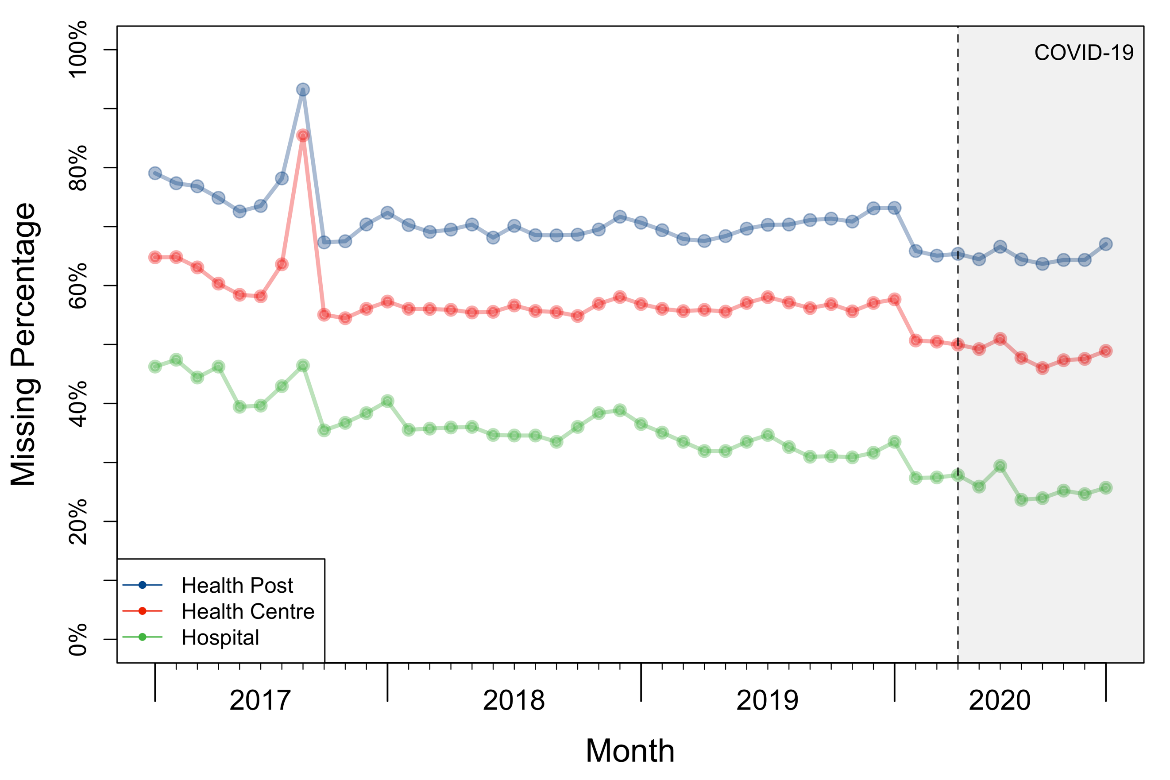


Additional file 1: Figure S.4a: Missing percentages for vaccinations (DTP, BCG, OPV, and PVC-13)


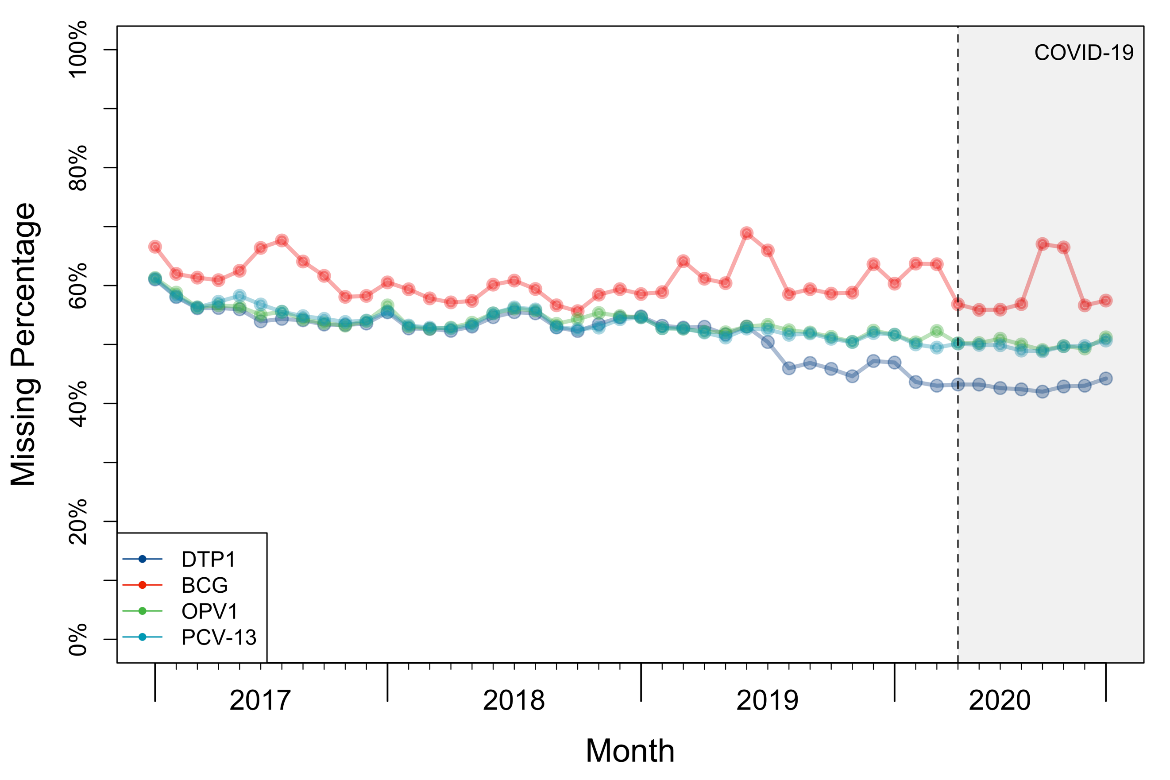


Additional file 1: Figure S.4b: Missing percentages for DTP vaccination visits by facility level


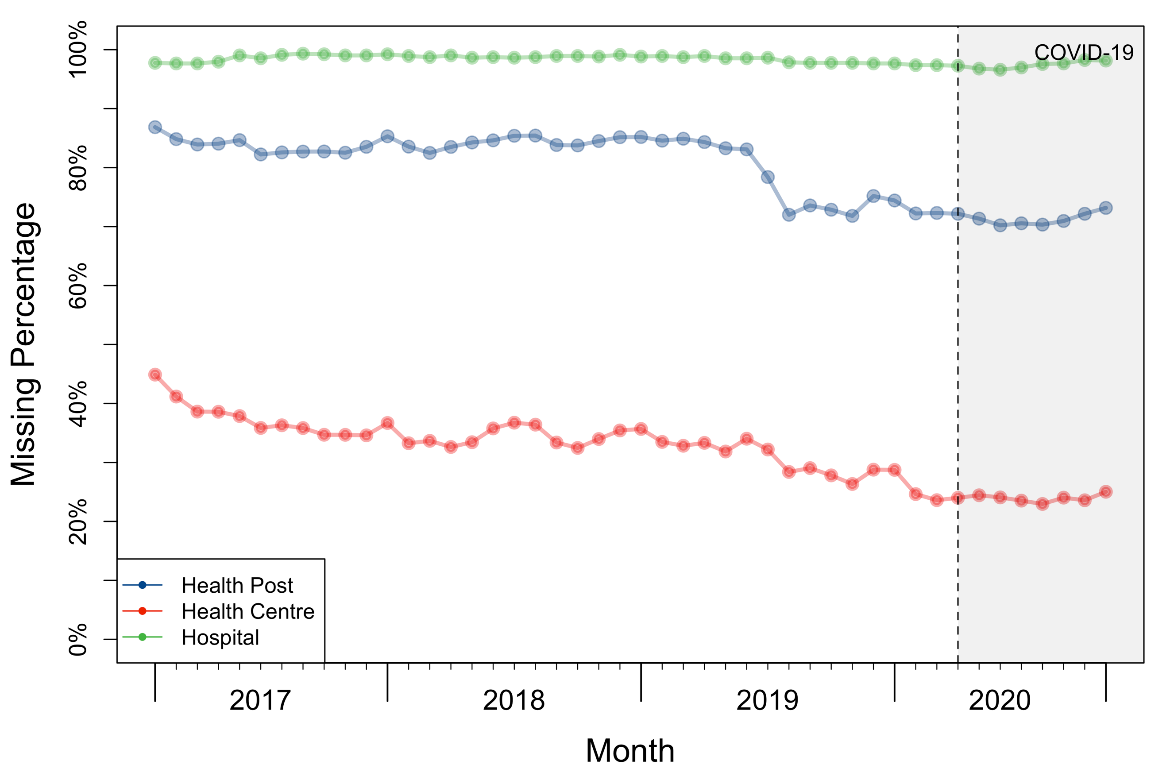


**Estimated Coefficients Under MAR Assumption**

Additional file 1: Figure S.5a: Estimated coefficients with 95% C.I.s for variable *Time* from linear regression


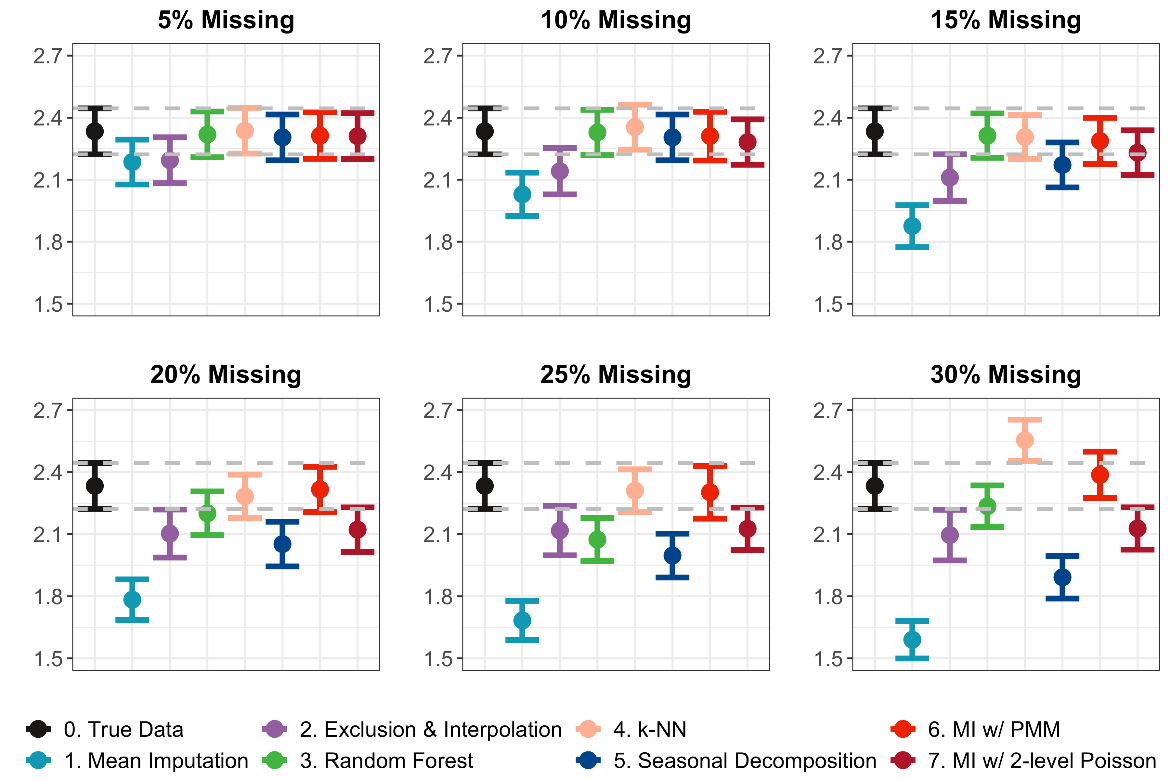


Additional file 1: Figure S.5b: Estimated coefficients with 95% C.I.s for variable *log(Population)* from linear regression


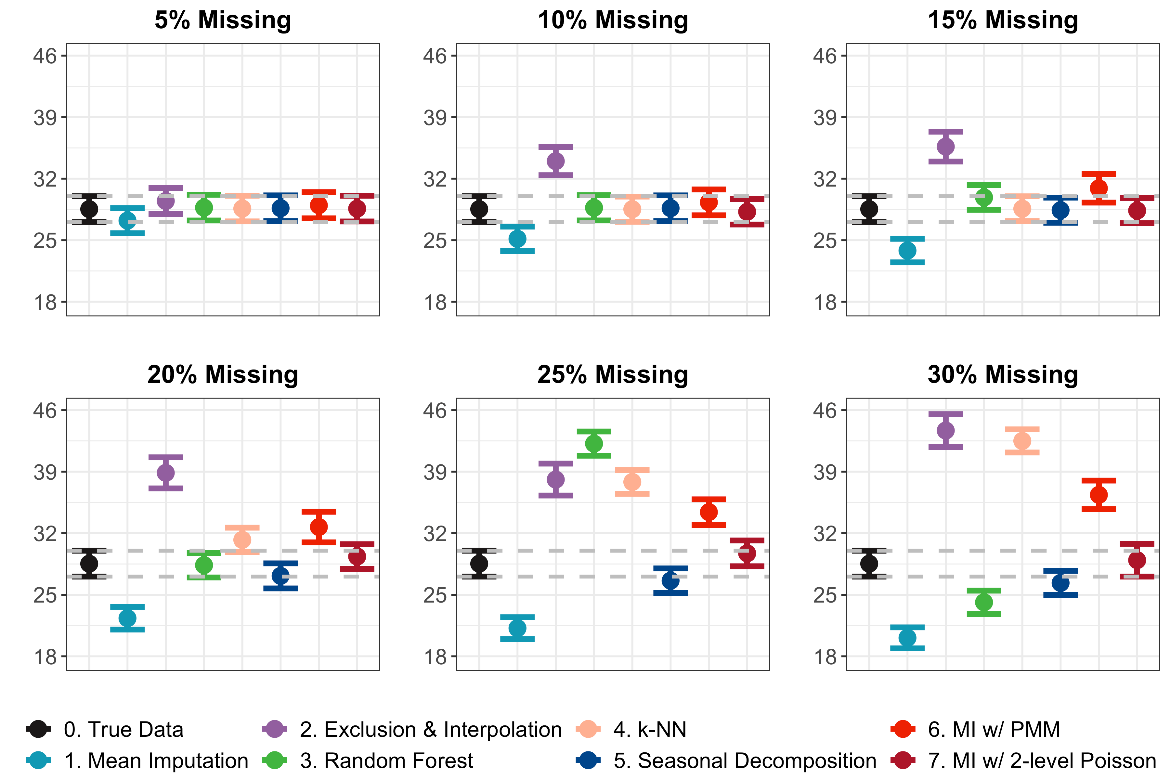


Additional file 1: Figure S.5c: Estimated coefficients with 95% C.I.s for the binary variable *COVID* from linear regression


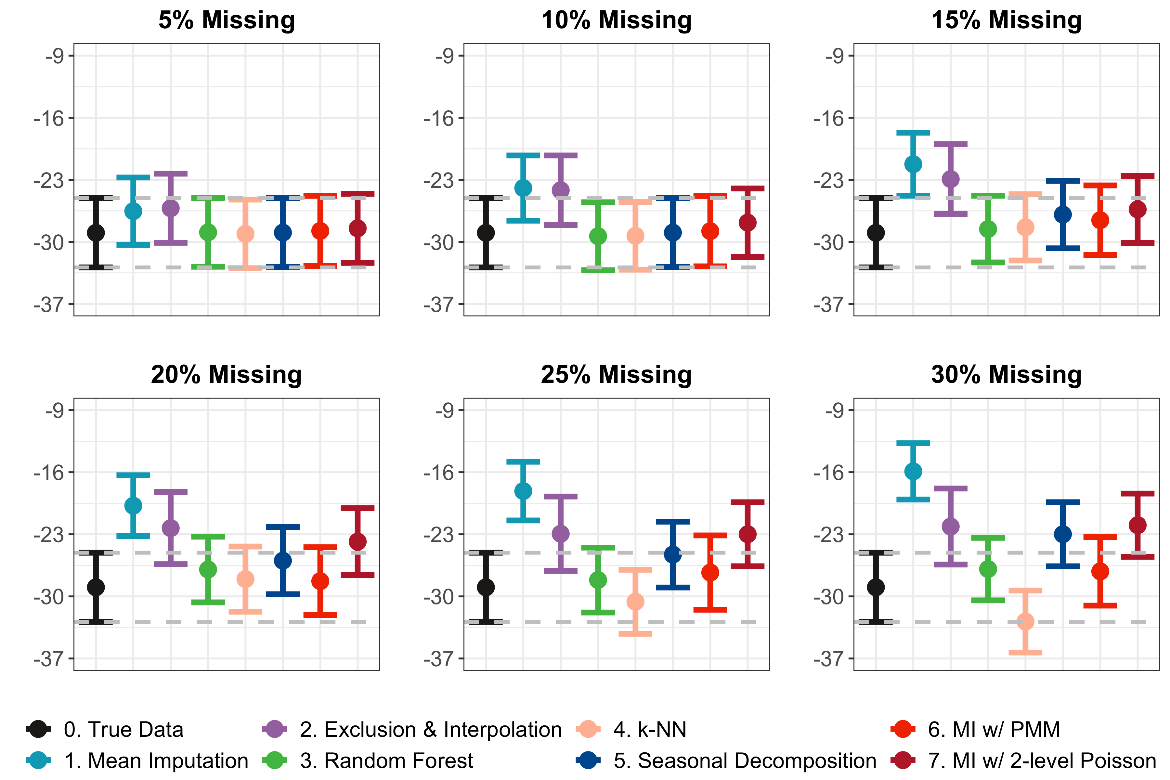


Additional file 1: Figure S.5d: Estimated coefficients with 95% C.I.s for variable *Facility Type: Hospital* from linear regression


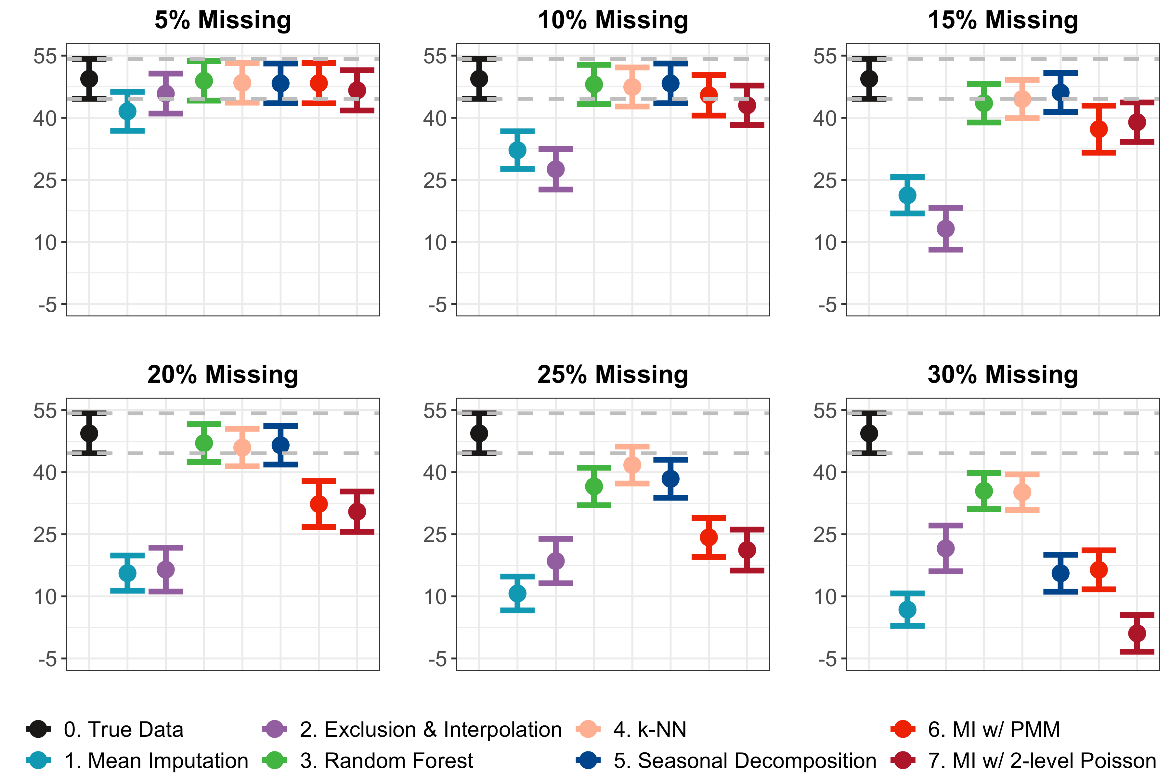


Additional file 1: Figure S.5e: Estimated coefficients with 95% C.I.s for variable *Facility Type: Health Post* from linear regression

**
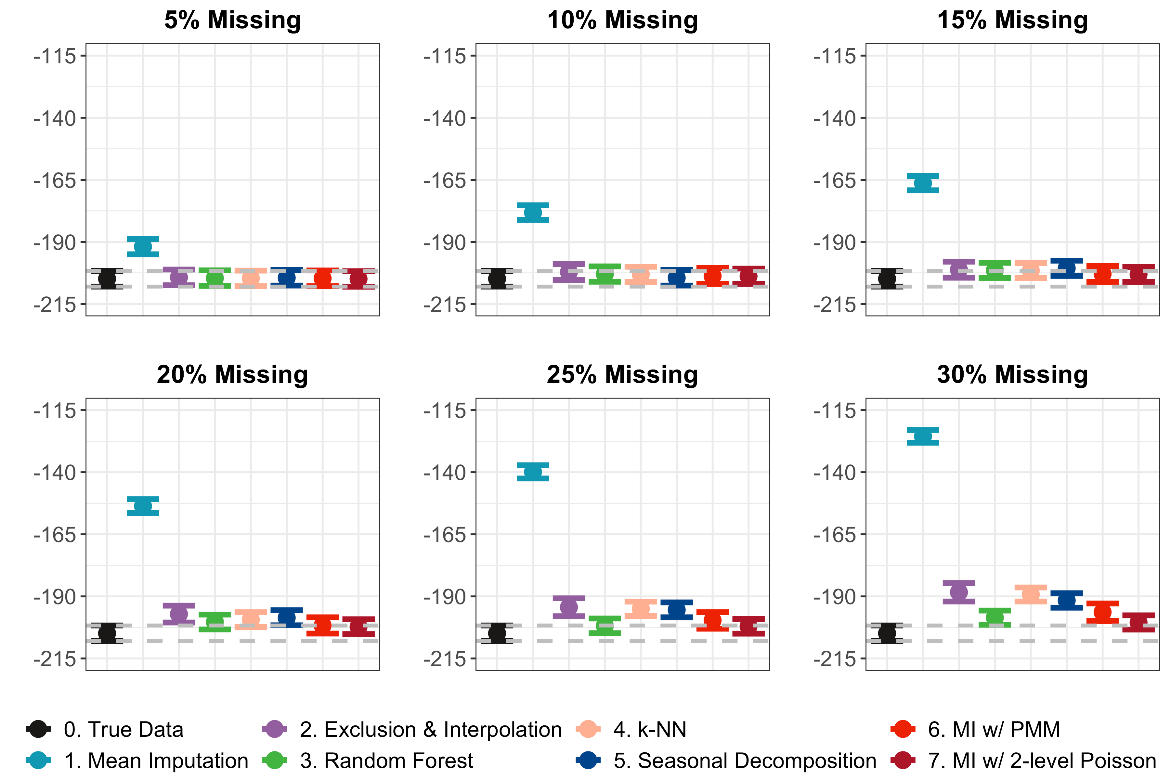
**

**Consecutive Missingness and Stability**

Additional file 1:
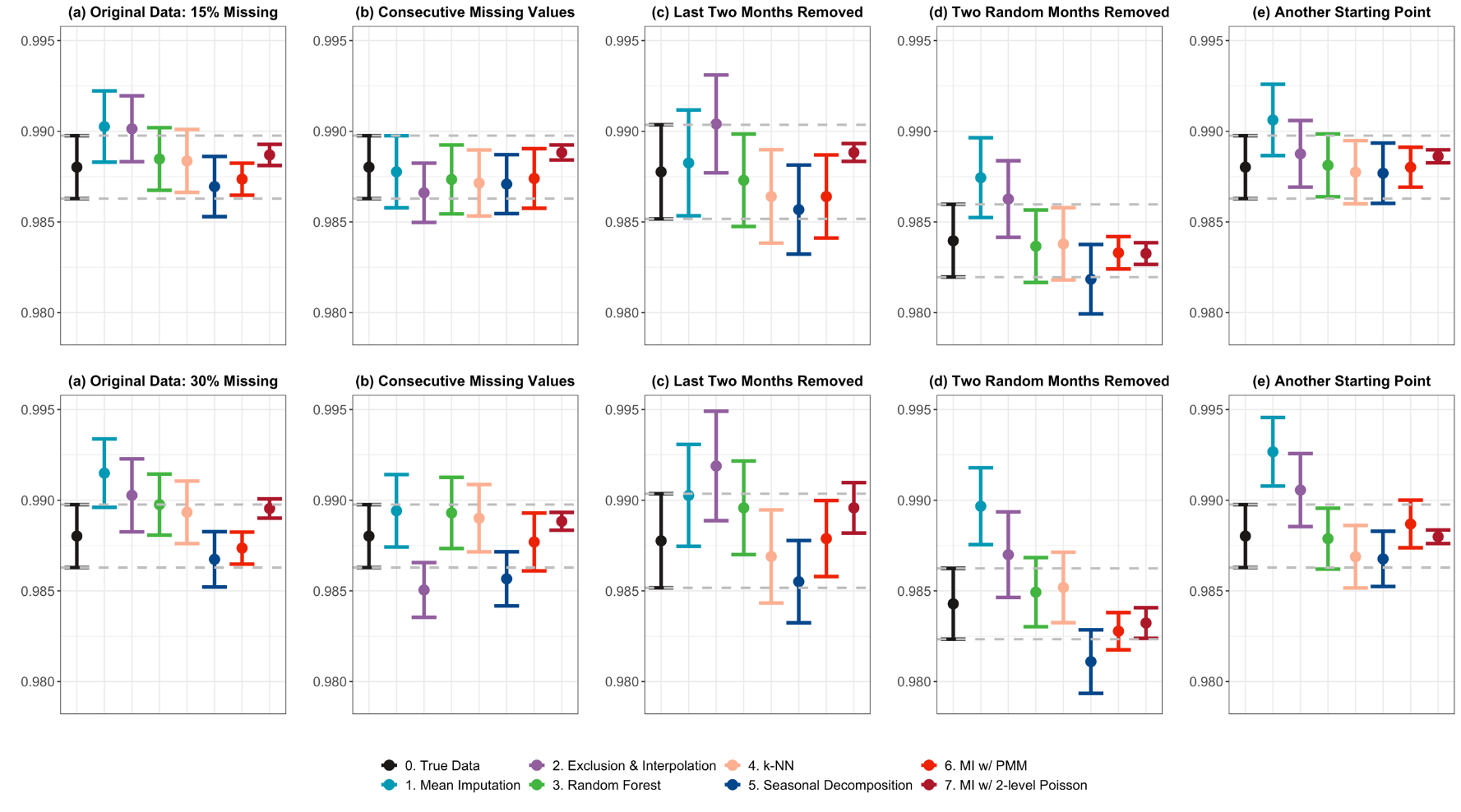
Figure S.6: Estimated trend change IRRs and 95% C.I.s with missing values inserted under different scenarios

**Pre-post Comparison**

**Paired t-test**

As shown in Additional file 1: Table S.1, all methods except mean imputation have returned a p-value less than 0.05, agreeing with the true result that one would have obtained using the complete data.

Additional file 1: Table S.1: p-values obtained from comparing group means using paired t-tests

| **Imputation Method** | | **5% Missing** | **10% Missing** | **15% Missing** | **20% Missing** | **25% Missing** | **30% Missing** |
| --- | --- | --- | --- | --- | --- | --- | --- |
|  | **Complete Data** | 0.008 ^†^ | 0.008 ^†^ | 0.008 ^†^ | 0.008 ^†^ | 0.008 ^†^ | 0.008 ^†^ |
| **SI** | **Mean Imputation** | 0.044 * | 0.08 | 0.084 | 0.101 | 0.187 | 0.598 |
|  | **Exclusion & Interpolation** | 0.021 * | 0.096 | 0.118 | 0.162 | 0.103 | 0.058 |
|  | **Random Forest** | 0.005 ^†^ | 0.001 ^†^ | 0.003 ^†^ | 0.007 ^†^ | 0.001 ^†^ | 0.013 * |
|  | **k-NN** | 0.006 ^†^ | 0.008 ^†^ | 0.009 ^†^ | 0.005 ^†^ | < 0.001 ^†^ | < 0.001 ^†^ |
|  | **Seasonal Decomposition** | 0.001 ^†^ | 0.001 ^†^ | < 0.001 ^†^ | < 0.001 ^†^ | < 0.001 ^†^ | < 0.001 ^†^ |
| **MI** | **MI with PMM** | 0.009 ^†^ | 0.011 * | 0.025 * | 0.009 ^†^ | 0.038 * | 0.040 * |
|  | **MI with 2-level Poisson** | 0.006 † | 0.004 † | 0.007 † | 0.006 † | 0.007 † | 0.009 † |

† for p-value < 0.01, * for p-value <0.05, and no annotation for p-value greater than 0.05

**R Codes**

##############################################################################

### Load packages

library(data.table)

library(tidyverse)

library(zoo)

library(lubridate)

library(imputeTS)

library(doParallel)

library(missForest)

library(randomForest)

library(mice)

library(countimp)

library(DMwR2)

library(lme4)

##############################################################################

### self-defined functions

add_segments <- function(df) {

df[["cases.agg"]] <- round(df[["cases.agg"]])

df[["date"]] <- as.yearmon(as.character(df[["PERIOD"]]), "%Y %m")

df[["rmonth"]] <- month(df[["date"]])

df[["ryear"]] <- year(df[["date"]])

# index time from 0

df <- df %>% mutate(time=time-1)

df <- df %>%

group_by(facility_ID) %>%

mutate(level = ifelse(time %in% c(0:37), 0, 1),

trend = ifelse(time %in% c(0:37), 0, time-38),

month = as.factor(rmonth),

year = as.factor(ryear))

return(df)

}

fit_glmm <- function(df) {

glmm.fit <- glmer(cases.agg ~ time + level + trend

+ faclevel

+ month

+ log.pop

+ (1+time| facility_ID),

data=add_segments(df),

nAGQ = 0,

family=poisson(link = "log"))

return(glmm.fit) }

##############################################################################

# The codes on merging and aggregating the raw HMIS data files are not provided here, as the data are not available to the public.

##############################################################################

### Insert missing data

# Assume MAR

# Input: dataset_all, which is a cleaned dataset containing ALL facilities

# data_complete, which is a dataframe containing only those always-reporting facilities

# Output: data2, which is the dataset with the same facilities from data_complete, but with

# missing values inserted

# cases.agg is the column containing the number of outpatients (clinical) visits

m_tmp <- dataset_all %>% mutate(missing = ifelse(is.na(cases.agg), 1, 0))

# Create a logistic model that captures the patterns of the missingness from the original data

m_model <- glm(missing ~ time + factor(season) + covid + log.pop + factor(faclevel)

+ factor(province) + factor(health_zone),

data = m_tmp, family = binomial)

# Predict the chance of being missing in the complete dataset, using the patterns found in the

# entire dataset

data2 <- data_complete

data2$m_pct <- predict(m_model, data_complete, type="response")

# to calibrate a missing proportion of 10%

set.seed(123)

missing <- sapply(data2$m_pct, function(pct) {rbernoulli(1, pct*1.519)})

# introduce missing

data2$cases.agg[missing==1] <- NA

##############################################################################

### Missing handling

# 1. Mean Imputation

data_mean <- data2

data_mean$cases.agg[is.na(data_mean$cases.agg)] <- round(mean(data_mean$cases.agg, na.rm = T))

# 2. Exclusion - exclude facilities with more than 3 consecutive missing months,

# and fill the rest missing values with interpolation

data_exclude <- data2

data_exclude$m_cases <- 0

data_exclude$m_cases[is.na(data_exclude$cases)==TRUE] <- 1

setDT(data_exclude)[, cm_cases := ifelse(m_cases== 0, 0, cumsum(m_cases)), .(facility_ID, rleid(m_cases))]

data_exclude <- data_exclude %>%

group_by(province, health_zone, facility_ID) %>%

summarise(exclude = ifelse(max(cm_cases)>3, 1, 0))

# Interpolation on the remaining facilities

data_exclude <- data_exclude %>%

filter(exclude==0) %>%

group_by(facility_ID) %>%

mutate(cases.agg=na_interpolation(cases.agg))

# 3. Random Forest

data_rf <- subset(data2, select=c(cases.agg, time, covid, log.pop, season, province, faclevel))

data_rf <- data_rf %>%

group_by(facility_ID) %>%

mutate(cases.lag1 = shift(cases.agg, -1),

cases.lead1 = shift(cases.agg, 1))

cores <- 4

cl <- makeCluster(cores)

registerDoParallel(cores)

set.seed(123)

cases.rf.impute <- missForest(data_rf, maxiter = 20, ntree = 10, parallelize="forests")

stopCluster(cl)

data_rf <- data2

data_rf$cases.agg <- round(cases.rf.impute$ximp$cases.agg)

# 4. k-NN

data_knn <- subset(data2, select=c(cases.agg, time, covid, log.pop, season, province, faclevel))

data_knn <- data_knn %>%

group_by(facility_ID) %>%

mutate(cases.lag1 = shift(cases.agg, -1),

cases.lead1 = shift(cases.agg, 1))

set.seed(123)

data_knn.impute <- knnImputation(data_knn, k = 10, scale = T, meth = "weighAvg")

data_knn <- data2

data_knn$cases.agg <- round(data_knn.impute$cases.agg)

# 5. Seasonal Decomposition

data_seadec <- data2 %>%

group_by(province_ID) %>%

na_seadec(algorithm = "kalman", find_frequency=TRUE)

# 6. MICE

data_mice <- subset(data2, select=c(cases.agg, time, covid, log.pop, season, province, faclevel))

data_mice <- data_mice %>%

group_by(facility_ID) %>%

mutate(cases.lag1 = shift(cases.agg, -1),

cases.lead1 = shift(cases.agg, 1))

mi <- mice(data_mice, m=5, method="pmm", maxit = 10, seed=123)

# 7. two-level Poisson with MICE

data_mice_multilevel <- subset(data2, select=c(cases.agg, PERIOD, time, covid, log.pop, season,

province, facility_ID, faclevel))

data_mice_multilevel <- data_mice_multilevel %>%

group_by(facility_ID) %>%

mutate(cases.lag1 = shift(cases.agg, -1),

cases.lead1 = shift(cases.agg, 1))

data_mice_multilevel$province <- as.integer(as.factor(data_mice_multilevel$province))

ini <- countimp(data_mice_multilevel, maxit=0)

meth <- ini$method

meth[meth!=""] <- "2l.poisson"

pred <- ini$predictorMatrix

pred[, "PERIOD"] <- 0

pred["cases.agg", c("time", "covid", " facility_ID ")] <- c(2, 2, -2)

pred["cases.lag1", c("time", "covid", " facility_ID ")] <- c(2, 2, -2)

pred["cases.lead1", c("time", "covid", " facility_ID")] <- c(2, 2, -2)

mi_multilevel <- countimp(data_mice_multilevel, method=meth, predictorMatrix=pred, m=5, maxit=10)

##############################################################################

### ITS with mixed effect Poisson

glmm_true <- fit_glmm(data_complete)

glmm_mean <- fit_glmm(data_mean)

glmm_exclude <- fit_glmm(data_exclude)

glmm_rf <- fit_glmm(data_rf)

glmm_knn <- fit_glmm(data_knn)

glmm_seadec <-fit_glmm(data_seadec)

glmm_mice <- mice::complete(mi, action = "long", include = TRUE) %>%

mutate(PERIOD = sort(unique(data2$PERIOD))[time],

LEVEL_5_UID = rep(data2$LEVEL_5_UID, 6)) %>%

add_segments() %>%

as.mids() %>%

mice::complete("all") %>%

lapply(glmer, formula = cases.agg ~ time + level + trend + faclevel + month + log.pop + (1+time|LEVEL_5_UID), family=poisson(link = "log")) %>%

pool()

glmm_mice_multilevel <- mice::complete(mi_multilevel, action = "long", include = TRUE) %>%

add_segments() %>%

as.mids() %>%

mice::complete("all") %>%

lapply(glmer, formula = cases.agg ~ time + level + trend + faclevel + month + log.pop + (1+time|LEVEL_5_UID), family=poisson(link = "log")) %>%

pool()
